# Preventive Effects of* Houttuynia cordata* Extract for Oral Infectious Diseases

**DOI:** 10.1155/2016/2581876

**Published:** 2016-06-20

**Authors:** Yasuko Sekita, Keiji Murakami, Hiromichi Yumoto, Takashi Amoh, Natsumi Fujiwara, Shohei Ogata, Takashi Matsuo, Yoichiro Miyake, Yoshiki Kashiwada

**Affiliations:** ^1^Department of Pharmacognosy, Institute of Biomedical Sciences, Tokushima University Graduate School, 1-78-1 Shou-machi, Tokushima 770-8505, Japan; ^2^Department of Oral Microbiology, Institute of Biomedical Sciences, Tokushima University Graduate School, 3-18-15 Kuramoto-cho, Tokushima 770-8504, Japan; ^3^Department of Conservative Dentistry, Institute of Biomedical Sciences, Tokushima University Graduate School, 3-18-15 Kuramoto-cho, Tokushima 770-8504, Japan

## Abstract

*Houttuynia cordata* (HC) (Saururaceae) has been used internally and externally as a traditional medicine and as an herbal tea for healthcare in Japan. Our recent survey showed that HC poultice (HCP) prepared from smothering fresh leaves of HC had been frequently used for the treatment of purulent skin diseases with high effectiveness. Our experimental study also demonstrated that ethanol extract of HCP (eHCP) has antibacterial, antibiofilm, and anti-inflammatory effects against* S. aureus* which caused purulent skin diseases. In this study, we focused on novel effects of HCP against oral infectious diseases, such as periodontal disease and dental caries. We determined the antimicrobial and antibiofilm effects of water solution of HCP ethanol extract (wHCP) against important oral pathogens and investigated its cytotoxicity and anti-inflammatory effects on human oral epithelial cells. wHCP had moderate antimicrobial effects against some oral microorganisms and profound antibiofilm effects against* Fusobacterium nucleatum*,* Streptococcus mutans*, and* Candida albicans*. In addition, wHCP had no cytotoxic effects and could inhibit interleukin-8 and CCL20 productions by* Porphyromonas gingivalis* lipopolysaccharide-stimulated human oral keratinocytes. Our findings suggested that wHCP may be clinically useful for preventing oral infectious diseases as a mouthwash for oral care.

## 1. Introduction

It has been considered that biofilms provide an ideal environment for microbial colonization and growth, because they limit the diffusion of antimicrobial agents and antiseptics and protect pathogens against the host immune attack [1, 2]. Therefore, dental biofilm forming in the oral cavity plays critical roles in the pathogenesis of oral infectious diseases such as dental caries and periodontal disease [3]. Moreover, oral microorganisms have been recently recognized as pathogens that can cause systemic chronic or severe infectious diseases [4, 5]. In particular, our previous report showed that a higher prevalence of* Candida* spp. as well as* Pseudomonas aeruginosa* and* Staphylococcus* spp. was observed in patients with cerebrovascular infarction and dysphagia [6]. Predominant fungi in humans such as* Candida albicans* can cause a variety of infectious diseases ranging from denture stomatitis to life-threatening invasive infections such as aspiration pneumonia, especially in immunocompromised or elderly patients [6–11]. Therefore, the reduction in adherence and biofilm formation by oral microorganisms contributes to preventing not only chronic oral infections but also potentially severe, systemic opportunistic infectious diseases, especially in elderly people.

Oral keratinocytes play an important role as the first physical barrier to bacterial invasion and organize the local innate immune system against colonizing microorganisms. In addition, they secrete proinflammatory mediators, such as chemokines and cytokines, in response to various stimuli including microbial infection and chemical irritation [12–14]. Lipopolysaccharide (LPS) from Gram-negative bacteria such as* Porphyromonas gingivalis*, which is a primary periodontal pathogen, upregulates the production of various proinflammatory mediators, such as interleukin- (IL-) 8 and CCL20, via signal cascades in the gingival epithelium [13, 15–20].

*Houttuynia cordata* Thunberg (HC) is a flowering perennial herb of the Saururaceae family and is commonly used as a health-promoting herbal tea in Japan. Our recent survey showed that HC poultice (HCP) prepared from smothering fresh leaves of HC in hot ashes or over charcoal fire wrapped with big leaves such as Japanese butterbur or aluminum foil had been frequently used for the treatment of purulent skin diseases with high effectiveness [21]. Our experimental study also demonstrated the anti-infective properties of HCP ethanol extract (eHCP), such as the antibacterial, bacteriostatic, and antibiofilm effects against* S. aureus* including methicillin-resistant* S. aureus* (MRSA) [21] which caused purulent skin diseases including furuncles [22, 23]. Moreover, we elucidated that eHCP has the anti-inflammatory effect to inhibit IL-8 and CCL20 productions from* S. aureus* lipoteichoic acid (LTA; TLR-2 ligand) stimulated human keratinocytes without any cytotoxic effect [21]. These findings suggested that HCP might be therapeutically useful as both antibiotic and anti-inflammatory modulators for skin infection and inflammation, such as purulent skin diseases including furuncle, with high and long-term effectiveness due to various infection-fighting properties of HCP.

However, there are no reports on the biological effects of HCP against oral infectious diseases. Hence, we focused on the novel effects of HCP against oral infectious diseases elicited by oral microorganisms. In this study, we first determined antimicrobial and antibiofilm effects of a water solution of HCP ethanol extract (wHCP), which might be used safely compared to eHCP. Next, we investigated these effects of wHCP against oral microorganisms including* Streptococcus mutans*, a primary dental caries-causing bacterium, and* Fusobacterium nucleatum* and* P. gingivalis*, primary periodontal pathogens, and* C. albicans*, a primary oral mycosis-causing fungus. We further examined cytotoxicity and anti-inflammatory effects of wHCP on human oral keratinocytes stimulated with* P. gingivalis* LPS as well as* S. aureus* LTA.

## 2. Materials and Methods

### 2.1. Plant Materials and Sample Preparation

HC used in this study was collected in Kochi City and identified by Dr. K. Fujikawa of the Kochi Prefectural Makino Botanical Garden, and voucher specimens (FOS-007536 and FOS-007537) were deposited there.

### 2.2. Preparations of Water Solution of* H. cordata* Poultice Ethanol Extract (wHCP)

wHCP was prepared as follows: approximately 20 g of fresh leaves of HC wrapped in aluminum foil was heated at 450 W for 5 min using an IH cooker to obtain HCP. This was shaken with 5 mL of ethanol for 10 min and then centrifuged for 20 min at 1,500 g. After centrifugation, the clear top layer was recovered to obtain eHCP and was stored at −20°C until wHCP preparation. After 100 *μ*L of eHCP was concentrated by drying under reduced pressure for 1 h (WKN-PV-1200, WAKENYAKU, Kyoto, Japan), the dried material was weighed and then dissolved in 100 *μ*L of sterile purified water to finally obtain wHCP.

### 2.3. Bacterial Strains and Growth Conditions

The bacterial strains used in this study are shown in Table 1.* Escherichia coli* and* Pseudomonas aeruginosa* were grown in Muller-Hinton broth (MHB, Becton Dickinson, Sparks, MD, USA) supplemented with 50 *μ*g mL^−1^ of CaCl_2_ and 25 *μ*g mL^−1^ of MgCl_2_, and methicillin-resistant* S. aureus* (MRSA) strains were grown in MHB supplemented with 25 *μ*g mL^−1^ of CaCl_2_, 12.5 *μ*g mL^−1^ of MgCl_2_, and 2% NaCl [24].* Streptococcus* spp. were grown in brain-heart infusion (BHI, Becton Dickinson) anaerobically.* C. albicans* was grown in Sabouraud dextrose medium composed of 10 g L^−1^ of peptone and 40 g L^−1^ of glucose aerobically.* F. nucleatum* and* P. gingivalis* were grown in BHI supplemented with 5 *μ*g mL^−1^ of hemin and 0.5 *μ*g mL^−1^ of menadione anaerobically.* Aggregatibacter actinomycetemcomitans* was grown in Todd Hewitt Broth (OXOID Ltd., Hampshire, UK) anaerobically. For the biofilm formation assays of* F. nucleatum*,* S. mutans*, and MRSA T31, Trypticase soy broth (TSB, Becton Dickinson) supplemented with 5 *μ*g mL^−1^ of hemin and 0.5 *μ*g mL^−1^ of menadione, TSB supplemented with 0.3% sucrose, and TSB supplemented with 0.3% glucose were used, respectively. For biofilm formation assay of* C. albicans*, yeast nitrogen base medium at pH 7 containing 2.5 mmol L^−1^ of N-acetylglucosamine (YNBNP) [25] was used.

### 2.4. Susceptibility Assay

The minimum inhibitory concentration (MIC) of wHCP was determined using a microbial broth dilution method. Approximately 10^6^ colony forming units (CFU) mL^−1^ of bacterial culture was inoculated into 100 *μ*L of medium containing a twofold serial dilution of wHCP in a 96-well culture plate (TPP, Trasadingen, Switzerland) and incubated anaerobically for* Streptococcus* spp.,* A. actinomycetemcomitans*,* F. nucleatum*, and* P. gingivalis* or aerobically for MRSA strains,* P. aeruginosa*,* E. coli*, and* C. albicans*, for 20 h or 48 h at 37°C. The MIC was defined as the lowest concentration showing no bacterial growth.

### 2.5. Biofilm Formation Assay

A crystal violet biofilm formation assay was performed to quantify the biofilm mass as described previously [26]. 2 *μ*L of MRSA T31,* S. mutans* MT8148, or* C. albicans* CAD1 at stationary phase or 5 *μ*L of* F. nucleatum* JCM8532 at stationary phase suspended in 150 *μ*L of broth or medium as described above was transferred in a U-bottomed 96-well plate (Cellstar, Greiner-Bio-One, Frickenhausen, Germany), and then wHCP (1%, 5%, or 10%) was added. Bacterial suspensions were incubated anaerobically for* F. nucleatum* JCM8532 and* S. mutans* MT8148 or aerobically for MRSA T31 and* C. albicans* CAD1 at 37°C for 6 or 24 h. Distilled H_2_O (1%, 5%, or 10%) was used as a negative control. After incubation, the biofilms formed were washed with purified water twice without disturbing the adherent biofilm, stained with 150 *μ*L of 0.1% crystal violet for 10 min at room temperature, and excess stain was removed by gentle washing with purified water twice. After drying, the stained biofilm was extracted from the well by adding 150 *μ*L of ethanol, and the absorbance of the extract from stained biofilm was measured at 595 nm using a microplate reader (model 680; Bio-Rad Laboratories, Hercules, CA, USA).

### 2.6. Cell Culture

RT-7 cells, an immortalized human oral keratinocyte cell line, kindly provided by Dr. N. Kamata (Hiroshima University, Hiroshima, Japan) as described previously [27], were cultured in Keratinocyte-SFM (Gibco BRL, Gaithersburg, MD, USA) supplemented with 100 U mL^−1^ of penicillin and 100 *μ*g mL^−1^ of streptomycin (Gibco BRL) at 37°C in a water-saturated atmosphere of 95% air and 5% CO_2_. Confluent monolayers were cultured with 1 *μ*g mL^−1^ of purified* S. aureus* LTA (InvivoGen, San Diego, CA, USA) or* P. gingivalis* LPS (InvivoGen) and/or 0.1%, 0.5%, or 1% wHCP.

### 2.7. Lactate Dehydrogenase (LDH) Cytotoxicity Assay

The effect of wHCP on cell cytotoxicity was determined using a lactate dehydrogenase (LDH) assay. Confluent RT-7 cell monolayers in a 24-well plate were cultured in Keratinocyte-SFM medium supplemented with 0.1%, 0.5%, 1%, or 10% wHCP for 24 h at 37°C. As a positive control, RT-7 cells were treated with 0.1% Triton X-100 for 10 min at room temperature. For the cytotoxicity assay, the levels of LDH released into the recovered cell culture supernatants were determined using an LDH cytotoxicity assay kit (Cayman Chemical, Ann Arbor, MI, USA) in accordance with the manufacturer's instructions. Absorbance was measured at 490 nm using a microplate reader (Bio-Rad Laboratories).

### 2.8. Enzyme-Linked Immunosorbent Assay (ELISA)

Enzyme-linked immunosorbent assay (ELISA) kits were used to quantify IL-8 and CCL20/macrophage inflammatory protein-3*α* (R&D Systems, Minneapolis, MN, USA) in cell culture supernatants.

### 2.9. Statistical Analysis

All statistical analyses were performed using the unpaired Student's *t*-test. Differences were considered significant when the probability value was less than 5% (*P* < 0.05).

## 3. Results

Recently, we demonstrated that eHCP showed antibacterial effects against MRSA [21]. However, considering the use of HCP for oral care, ethanol in eHCP should be substituted for other solvents, since it was cautioned in a previous review article that ethanol in mouthwashes has potentially an increased risk of oral cancer [28]. Therefore, we substituted ethanol with water in eHCP and first confirmed the antimicrobial effects of wHCP against various microorganisms including MRSA and oral pathogens. As shown in Table 2, wHCP had moderate antimicrobial effects on MRSA T31, MRSA COL,* S. mutans* MT8148,* S. oralis* ATCC10557,* F. nucleatum* JCM8532, and* P. gingivalis* ATCC33277 (MIC; 0.3–5.0%, 68–1085 *μ*g mL^−1^).

**Figure 1:**
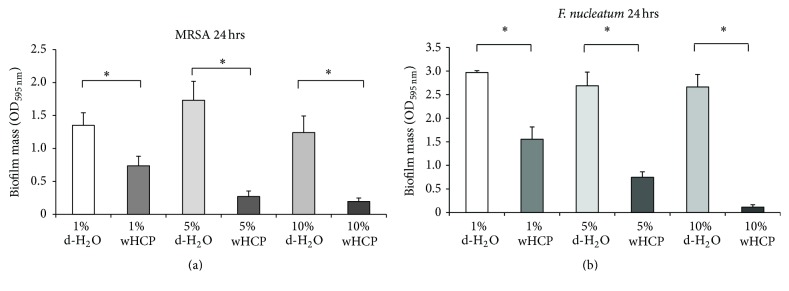
Antibiofilm effects of water solution of* Houttuynia cordata* poultice ethanol extract (wHCP) on 24 h biofilm formations by MRSA T31 (a). Antibiofilm effects of water solution of* Houttuynia cordata* poultice ethanol extract (wHCP) on 24 h biofilm formations by* F. nucleatum* (b). Distilled H_2_O (1%, 5%, or 10%) was used as a negative control. ^*∗*^Significant differences between the indicated groups at *P* < 0.001 using Student's *t*-test.

**Figure 2:**
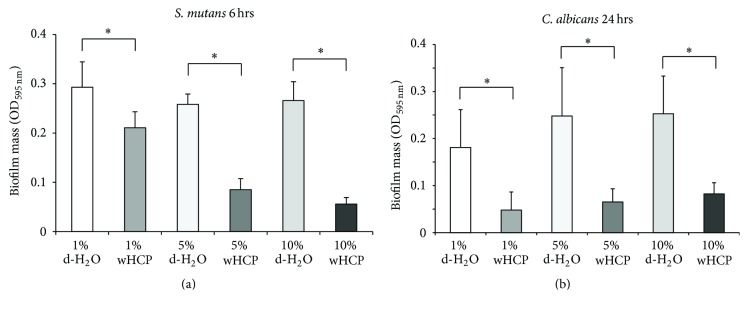
Antibiofilm effects of water solution of* Houttuynia cordata* poultice ethanol extract (wHCP) on 6 h biofilm formation by* S. mutans* MT8148 (a). Antibiofilm effects of water solution of* Houttuynia cordata* poultice ethanol extract (wHCP) on 24 h biofilm formations by* Candida albicans* CAD1 (b). Distilled H_2_O (1%, 5%, or 10%) was used as a negative control. ^*∗*^Significant differences between the indicated groups at *P* < 0.001 using Student's *t*-test.

We then examined the antibiofilm effects of wHCP for* F. nucleatum*,* S. mutans*, and* C*.* albicans*, which form biofilms in human oral cavity including denture surface [29, 30], as well as MRSA. For this experiment, we used the culture of MRSA T31 clinical isolate because MRSA COL had less biofilm formation activity (data not shown). A biofilm formation assay revealed that wHCP significantly inhibited 24 h biofilm formation by MRSA T31 and 24 h biofilm formation by* F. nucleatum* in a concentration-dependent manner (Figures 1(a) and 1(b)). The biofilm formation assay also revealed that wHCP significantly inhibited 6 h biofilm formation by* S. mutans* in a concentration-dependent manner (Figure 2(a)). Moreover, the biofilm formation assay with* C. albicans* revealed that wHCP (1%, 5%, and 10%) reduced 24 h biofilm formation (Figure 2(b)). In each experiment, 10% wHCP could not affect the growth of these microorganisms (data not shown). These findings revealed that wHCP had antibiofilm activity against major oral pathogens as well as MRSA.

**Figure 3:**
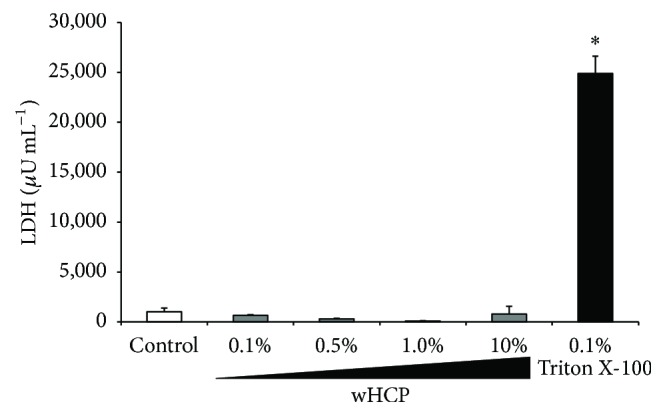
No cytotoxic effect of water solution of* Houttuynia cordata* poultice ethanol extract (wHCP) on oral keratinocytes. As a positive control, RT-7 cells were treated with 0.1% Triton X-100 and shaken gently for 10 min at room temperature. ^*∗*^Significant differences versus control group at *P* < 0.001 using Student's* t*-test.

To confirm the absence of any cytotoxic effect of wHCP on keratinocytes, we measured the level of LDH released from RT-7 cells. As a positive control, keratinocytes were treated with 0.1% Triton X-100 for 10 min. As shown in Figure 3, no cytotoxic effect of wHCP (up to 10%) was observed. These results indicated that wHCP may be clinically used for oral applications.

**Figure 4:**
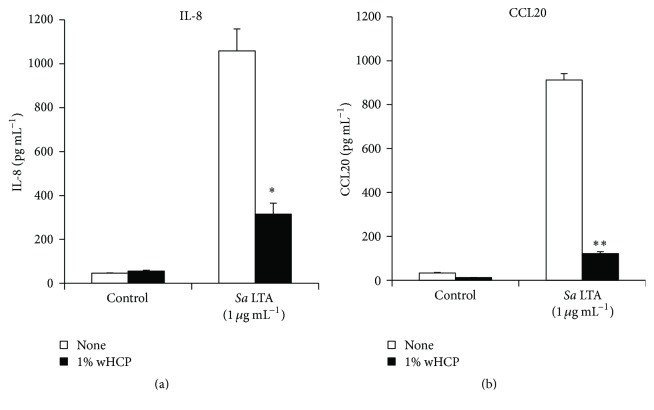
Inhibitory effect of water solution of* Houttuynia cordata* poultice ethanol extract (wHCP) on IL-8 (a) and CCL20 (b) productions in oral keratinocytes stimulated with* S. aureus* LTA for 24 h. Significant differences versus control group at ^*∗*^ *P* < 0.05 and ^*∗∗*^ *P* < 0.01 using Student's *t*-test.

**Figure 5:**
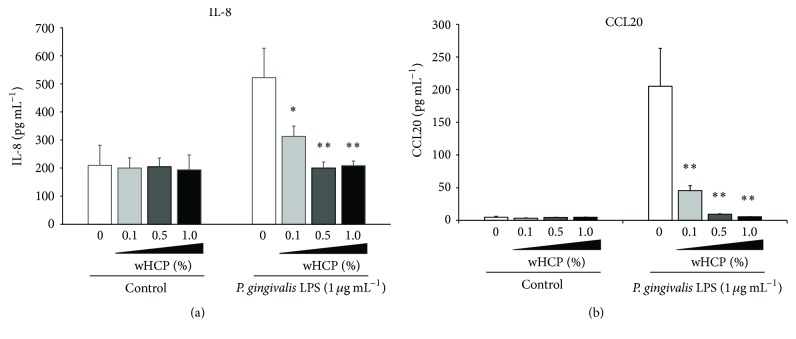
The inhibitory effects of water solution of* Houttuynia cordata* poultice ethanol extract (wHCP) on IL-8 (a) and CCL20 (b) productions in oral keratinocytes stimulated with* P*.* gingivalis *for 24 h. Significant differences versus control group at ^*∗*^ *P* < 0.05 and ^*∗∗*^ *P* < 0.01 using Student's *t*-test.

**Figure 6:**
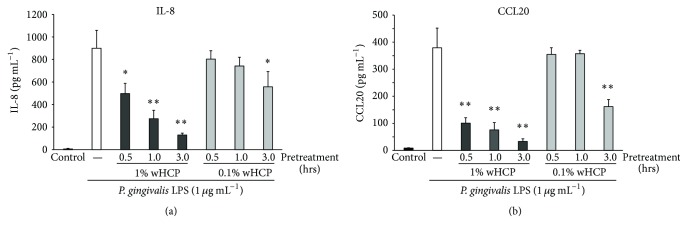
The inhibitory effects of water solution of* Houttuynia cordata* poultice ethanol extract (wHCP) pretreatment on IL-8 (a) and CCL20 (b) productions in oral keratinocytes stimulated with* P*.* gingivalis* for 24 h. Significant differences versus no pretreatment control group at ^*∗*^ *P* < 0.05 and ^*∗∗*^ *P* < 0.01 using Student's *t*-test.

In this study, we examined the inhibitory effects of wHCP on IL-8 and CCL20 productions in* S. aureus* LTA-stimulated or* P. gingivalis* LPS-stimulated RT-7 cells. At first, we confirmed that 1% wHCP significantly inhibited IL-8 and CCL20 productions in RT-7 cells stimulated with* S. aureus* LTA (Figure 4). We also found that wHCP significantly inhibited IL-8 and CCL20 productions in RT-7 cells stimulated with* P. gingivalis* LPS for 24 h in a concentration-dependent manner (Figure 5). However, it is unrealistic to apply wHCP in the mouth for long period. We next examined whether short-term pretreatment with wHCP could inhibit IL-8 and CCL20 productions in* P. gingivalis* LPS-stimulated RT-7 cells. IL-8 and CCL20 production in* P. gingivalis* LPS-stimulated RT-7 cells was significantly inhibited by 0.5 and 3 h pretreatments with 1% and 0.1% wHCP, respectively (Figure 6). These results suggested that wHCP may be clinically useful for preventing oral infectious diseases such as periodontal disease as mouthwash for oral care.

## 4. Discussion

In this study, wHCP was shown to have antimicrobial effects against various oral microorganisms, antibiofilm effects against MRSA,* S. mutans*,* F. nucleatum*, and* C. albicans*, and inhibitory effects on IL-8 and CCL20 productions by* S. aureus* LTA-stimulated or* P. gingivalis* LPS-stimulated human oral keratinocytes with no cytotoxic effect. These findings contributed to assessing the potential for wHCP as new oral care products.

It has been reported that HC contains various essential oils (terpenoids, hydrocarbons, esters, alcohols, ketones, aldehydes, acids, phenols, ethers, and mixed compounds) and flavonoids (quercitrin, isoquercitrin, afzelin, hyperin, and rutin) [31]. Among compounds contained in the essential oil, aldehydes such as lauryl aldehyde and decanoyl acetaldehyde (houttuynin) have antimicrobial effects against Gram-positive bacteria and antifungal effects [32–35]. Among these, decanoyl acetaldehyde has better antimicrobial activity than other aldehydes but shows lower stability [32]. Our present results demonstrated that wHCP contains higher levels of active constituents such as aldehydes because of our unique preparation methods by the combination of smothering and ethanol extraction.

Recent studies have reported that extracts from many plants have antimicrobial and/or antibiofilm effects against oral microorganisms including* S. mutans*,* S. sobrinus*,* P. gingivalis*, and* C. albicans* [36–39]. It has been also reported that HC has many activities such as diuretic, antibacterial, antiviral, anticancer, and antiallergic effects [31, 40]; however, there have not been any studies on its antimicrobial and/or antibiofilm effects against oral microorganisms and anti-inflammatory effects on oral keratinocytes. This study successfully demonstrated that wHCP has antimicrobial effects against various pathogens that normally colonize the oral cavity (Table 2) and antibiofilm effects against major oral pathogens,* F. nucleatum*,* S. mutans*, and* C. albicans* (Figures 1 and 2).

Our previous study demonstrated that CCL20 produced by inflamed gingival epithelial cells appears to be closely related to proinflammatory responses of the gingiva and plays an important regulatory role in specific lymphocyte migration into periodontal diseased tissue [18]. In addition, TLR2, a pattern recognition receptor for LTA from* S. aureus* or LPS from* P. gingivalis*, is prominently expressed in the pocket epithelium of periodontal tissues with chronic periodontitis and participated in the signaling cascade to upregulate the production of IL-8 [13, 15–17, 19, 20]. We demonstrated that wHCP significantly inhibited IL-8 and CCL20 productions from* P. gingivalis* LPS-stimulated oral keratinocytes in a concentration-dependent manner (Figure 5). Moreover, short-term pretreatment with wHCP significantly inhibited IL-8 and CCL20 productions in RT-7 cells stimulated with* P. gingivalis* LPS (Figure 6). Taken together, we inferred that wHCP has multiple activities on both oral microorganisms and host cells and may be clinically useful for the prevention of oral infectious diseases, including dental caries and periodontal diseases, as a mouthwash for oral care without cytotoxic effects. Essential oils from HC have anti-inflammatory effects mediated by the inhibition of cyclooxygenase-2 by a mechanism similar to that of nonsteroidal anti-inflammatory drugs [41] and also by inhibiting nitric oxide and tumor necrosis factor-*α* production in LPS-stimulated mouse peritoneal macrophages [42]. Among the flavonoid glycosides from HC, quercitrin (3-rhamnosylquercetin) had an inhibitory effect on acute inflammatory edema induced by various phlogistic agents, such as histamine and serotonin [43]. HC also inhibited LTA-induced inflammation in human dermal fibroblast [44]. These previous reports and our present results suggest that wHCP contained effective constituents involved in the host inflammatory response to infection.

Biofilm formed by* Candida* spp. can provide an attachment site for other microorganisms, but wHCP can inhibit biofilm formation by* C. albicans*; therefore, we suggest that wHCP might aid in preventing aspiration pneumonia.* F. nucleatum* and* P. gingivalis*, which are oral opportunistic bacteria, recently have been ascended to central players based on their participation in complex codependent mechanisms of systemic chronic diseases including cancer, rheumatoid-arthritis, and diabetes in terms of risk and pathogenesis [5]. According to our findings showing wHCP has moderate antimicrobial and antibiofilm effects on both* F. bacterium* and* P. gingivalis*, we infer that wHCP might reduce risk of progression to chronic systemic diseases from oral infection.

For oral application, smell and taste are very important factors, in contrast to external application for skin diseases. Because wHCP is bland and innocuous, it would be acceptable in this respect. Considering that oral care contributes to systemic health, we infer that clinical applications of wHCP may be effective for preventing oral infections, such as dental caries, periodontal diseases, and aspiration pneumonia in immunocompromised hosts and elderly people. Further studies are needed to characterize the constituents of wHCP, which exhibit antimicrobial, antibiofilm, and anti-inflammatory activities, and to clarify the regulatory mechanisms against oral microorganisms such as* S. mutans*,* F. nucleatum*,* P. gingivalis*, and* C. albicans*.

## 5. Conclusions

This study demonstrated the anti-infective properties of wHCP, including antimicrobial effects against some oral pathogens and antibiofilm effects against* F. nucleatum*,* S. mutans*, and* C. albicans*. Moreover, we elucidated that wHCP had no cytotoxic effects and inhibitory effects on IL-8 and CCL20 production by* P. gingivalis* LPS-stimulated human oral keratinocytes. wHCP, which is bland and innocuous, may be clinically useful for preventing oral infectious diseases, including dental caries, periodontal diseases, aspiration pneumonia, denture stomatitis, and oral candidiasis, as a mouthwash for oral care.

## Figures and Tables

**Table 1:** Bacterial strains.

Strain	Source
MRSA T31	Clinical isolate
MRSA COL	Type strain
*Streptococcus mutans* MT8148	Clinical isolate
*Streptococcus mutans* UA159	Clinical isolate
*Streptococcus sobrinus* 1310	Clinical isolate
*Streptococcus gordonii* ATCC10558	Type strain
*Streptococcus oralis* ATCC10557	Type strain
*Streptococcus constellatus* 4528	Clinical isolate
*Streptococcus intermedius* 40138	Clinical isolate
*Aggregatibacter actinomycetemcomitans* Y4	Wild type
*Fusobacterium nucleatum *JCM8532	Wild type
*Porphyromonas gingivalis* ATCC33277	Type strain
*Pseudomonas aeruginosa* PAO1	Wild type
*Escherichia coli* K1	Wild type
*Candida albicans* CAD1	Clinical isolate

**Table 2:** MIC of wHCP.

Bacterial strain	MIC	
	(%)^*∗*^	(*μ*g mL^−1^)
MRSA T31	0.3	68
MRSA COL	1.25	271
*Streptococcus mutans* MT8148	5	1085
*Streptococcus mutans* UA159	>10	>2170
*Streptococcus sobrinus* 1310	>10	>2170
*Streptococcus gordonii* ATCC10558	>10	>2170
*Streptococcus oralis* ATCC10557	1.25	271
*Streptococcus constellatus* 4528	>10	>2170
*Streptococcus intermedius* 40138	>10	>2170
*Aggregatibacter actinomycetemcomitans* Y4	>10	>2170
*Fusobacterium nucleatum *JCM8532	2.5	543
*Porphyromonas gingivalis* ATCC33277	1.25	271
*Pseudomonas aeruginosa* PAO1	>10	>2170
*Escherichia coli* K1	>10	>2170
*Candida albicans* CAD1	>10	>2170

^*∗*^The concentration of wHCP in medium (V/V).
